# Evaluation of Proanthocyanidin-based dentifrices on dentin-wear after erosion and dental abrasion - *In situ* study

**DOI:** 10.4317/jced.59071

**Published:** 2022-04-01

**Authors:** Tamires-de Luccas Bueno, Tamires-Alves-Pereira da Silva, Fabio-Antonio-Piola Rizzante, Ana-Carolina Magalhães, Daniela Rios, Heitor-Marques Honório

**Affiliations:** 1 DDS, Ms, PhD. Department of Operative Dentistry, Endodontic and Dental Materials, Bauru School of Dentistry, University of São Paulo-USP, Bauru, SP, Brazil; 2 DDS, Surgeon Dentist; 3 DDS, MSc, PhD, MBA. Associate Professor and Director of Research Department of Oral Rehabilitation Medical University of South Carolina, James B. Edwards College of Dental Medicine, Charleston, SC, USA; 4 DDS, Ms, PhD. Biochemistry, Department of Biological Sciences Bauru School of Dentistry-USP; 5 DDS, M.D, PhD. Associate Professor, Discipline of Pediatric Dentistry, Bauru School of Dentistry, University of São Paulo- USP, Department of Pediatric Dentistry, Orthodontics and Public Health; 6 DDS, M.D, PhD. Associate Professor, Discipline of Research Methodology and Statistics, Bauru School of Dentistry, University of São Paulo- USP, Department of Pediatric Dentistry, Orthodontics and Public Health

## Abstract

**Background:**

Proanthocyanidin has been considered as a preventive agent against erosion because of its properties, which involves remineralization, reduction of demineralization and matrix metalloproteinases (MMPs) inhibition. Thus, the aim of this in situ study was to evaluate the effect of proanthocyanidin-based dentifrices on wear resistance of dentin specimens subjected to erosion associated with abrasion.

**Material and Methods:**

This crossover double-blinded study was performed in 5 phases of 5 days each, with 10 healthy volunteers who wore 5 palatal devices (1 for each phase) with 4 dentin specimens. The groups under study were: G1 – placebo dentifrice (negative control group); G2 – 0.012% chlorhexidine dentifrice (1st positive control group); G3 – NaF 1110 ppm fluoride dentifrice (2nd positive control group); G4 – 10% purified proanthocyanidin dentifrice (1st test group); G5 – fluoride + proanthocyanidin dentifrice (2nd test group). Erosion was performed by palatal device immersion in acid beverage (Coca-cola®), 3 times daily for 5 minutes during 5 days. Abrasion was applied after the first and third erosive cycles by using a eletric toothbrush during 15 seconds in each specimen with the application of the studied dentifrices slurry. The response variable was depth of dentin loss (µm) measured by profilometry. Data were analyzed by Repeated Measures Analysis of Variance followed by LSD Fisher’s test (*p*<0.05).

**Results:**

Data (G1: 1.76 ± 0.55A; G2: 1.19 ± 0.42B; G3: 1.29 ± 0.34B; G4: 0.93 ± 0.38C; G5: 0.82 ± 0.34C) showed that G4 and G5 did not presented significant difference between them, but showed less dentine loss when compared to all other groups. G1 presented the highest dentin wear.

**Conclusions:**

Proanthocyanidin and the combination of proanthocyanidin and fluoride dentifrices revealed the best results, showing that these formulations could be a promising alternative for patients who suffer with dentin erosion.

** Key words:**Dentin, erosion, cross-liking agent, metalloproteinases, toothpastes.

## Introduction

Non carious lesions such as dental erosion have become more prevalent in dental offices as a result of increased life expectance and better maintenance of teeth in the oral cavity, associated with dietary habits/external acid (i.e. wide consumption of acidic foods and beverages), as well as stomach acids/internal acid, leading to tooth structure dissolution (1-3).

Usually, such lesions are recognized when the patient started to experience dentin hypersensitivity due to exposure of dentin in the oral cavity. The lesion’s rate of progression depends on frequency and duration of exposure, adjuvant factors such as dental abrasion resulting of toothbrush and/or parafunctional habits (1,2,4).

Non carious lesions in dentin tend to progress at a faster rate since dentin presents a higher critical pH (the dentin demineralizes at any pH below 5.5), lower surface hardness (when compared to enamel), presence of Matrix Metalloproteinases/MMPs and cysteine cathepsins which degrades the exposed collagen, contributing to dentin wear (5-8). Within this context, demineralized organic matrix (DOM) acts as a barrier for acid penetration, and has some capabilities in preventing the advance of non-carious lesions, including some resistance to abrasion (5,9).

Many products have been studied with goal of avoid the collapse of the DOM as well as increase its resistance to wear. Proanthocyanidins (PA) are found in natural products and many studies have shown its capacity of remineralization, decrease of demineralization, interaction and synthesis of collagen in dentin and increase in its mechanical proprieties (10,11). The PA interaction with collagen in dentin probably occurs due to chemical linkage, by hydrogen bonding between phenolic hydroxyl part of PA with the protein amide carbonyl of collagen generating a cross-linkers mechanism, but also by hydrophobic and covalent linkage (12). This component has been tested in different ways, concentrations and time of application and in all studies presented good results (13,14). The use of PA could contribute in the prevention of mineral loss, especially if it becomes widely available in products of daily use (i.e. dentifrices) (15).

Due to greater difficulty of quantity the mineral loss in clinical research, many *in vitro* and in situ studies are developed (16) with aim reduce the dental wear, find answers for solve dental erosion problem, and evaluate new products may be clinically used (3). However, in situ studies allow simulation inside of mouth, executed after *in vitro* studies, being considered an accurate methodology (16).

Based on the above considerations, the aim of this present study was to evaluate the effects of dentifrices containing PA on the dentin mineral loss after erosive and abrasive challenges, using in situ conditions. The null hypothesis tested was that PA dentifrices would not minimize dentin wear when compared with other tested dentifrices.

## Material and Methods

-Experimental design

Definition of sample size was through of pilot study in laboratory as well as for *in vitro* study previously performed.

**Figure 1:**
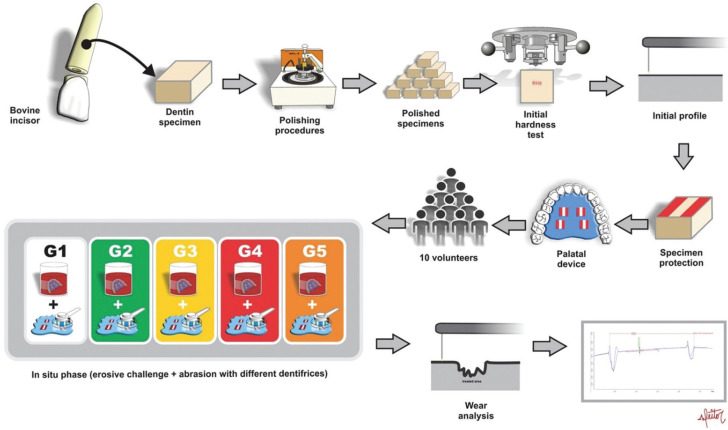
Flowchart of the study.

The present crossover, double-blinded, in situ study was performed in 5 phases of 5 days each, with 10 volunteers that wore 5 palatal devices (1 for each phase) with 4 dentin blocks each. The study factor was the dentifrices divided in 5 levels: G1 – placebo dentifrice (negative control group); G2 – 0.012% chlorhexidine dentifrice (1st positive control group); G3 – NaF 1110 ppm fluoride dentifrice (2nd positive control group); G4 – 10% purified proanthocyanidin dentifrice (1st test group); G5 – fluoride + proanthocyanidin dentifrice (2nd test group), having as response variable the depth of dentin loss (µm) measured using profilometry (Fig. 1).

For sample size calculation, considering the results of a pilot study, adopting 1,23µm as expected dentin loss standard deviation 1µm as a minimum detecTable difference in means between groups (Effect Size), a minimum sample size of 34 volunteers was set for this study (β error = 20% and α error = 5%). However, considering possible dropouts, there was a 20% increase in the sample of groups and 40 specimens were included in the study for each group, resulting in 10 volunteers.

-Specimens Preparation and Analysis

Two hundred dentin blocks (4×4×2mm) were extracted from bovine roots using 2 diamond discs (Extec Corp, Enfield, CT, USA) separated by a spacer of 4mm, adapted on an ISOMET low-speed saw machine (Buehler Ltd, Lake Bluff, IL, USA). Specimens were ground flat with 320, 600 and 1200 grit Al2O3 papers (Buehler) using a polishing machine (APL 4, Arotec, Cotia, SP, Brazil). Between each paper grit, samples were cleaned in ultrasonic T7 Thornton (Unique Ind. e Com. de produtos Eletrônicos Ltda., São Paulo, SP, Brasil) with deionized water for 2 minutes. Samples were stored in 0.1% thymol solution (pH 7.0) at 4°C until used.

After specimens’ preparation, Knoop microhardness was performed with 25g load for 10 seconds (HMV-2000; Shimadzu Corporation, Tokyo, Japan) in order to ensure similar initial conditions among all groups (range of values 32). All specimens were sterilized by gamma radiation, randomized and divided into 5 groups. Two reference marks were created on the dentin surface using a scalpel blade and an initial profilometry was performed (Mahr Perthometer, Göttingen, Germany) as follows: five measurements were made for each specimen at pre-established distances from the edge: 2.25; 2.0; 1.75; 1.5 and 1.25 µm. Thus, each one of the 200 specimens was measured five times. For each specimen, its final wear was the average of these five measurements. Two-thirds of the surface was covered with red nail varnish maintaining 2 mm of exposed dentin area in the center. During the pilot study, the reliability analysis of the profilometry measures was performed. Twenty specimens were subjected to an erosive challenge and wear was assessed at baseline and after 15 days. The intraclass correlation coefficient indicated a good reliability of the method (ICC= 0.83).

After approved by the local ethics committee (CAAE: #65055317.7.0000.5417) and conformed 1964 Helsinki Declaration for experiments involving humans, ten volunteers were selected based on the inclusion and exclusion criteria (Table 1). For each of the 10 volunteers, an alginate impression (Jeltrate, Dentisply/ Sirona, *Pi*rassununga, SP, Brazil) of the maxillary arch was performed using a stock metal tray, the casts were poured with type IV stone (Durone IV, Dentisply/ Sirona, Pirassununga, SP, Brazil) and the appliances were designed using self-curing acrylic resin (JET, Artigos Odontológicos Clássico Ltda, São Paulo, SP, Brazil) with 4 sites (4 x 4 x 3 mm) where the randomized specimens were positioned and fixed with wax.

**Table 1:**
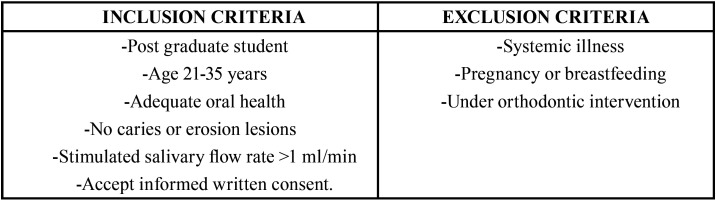
Criteria of inclusion and exclusion for selection of volunteers.

The ten volunteers wore 5 removable palatal appliances with 4 dentin specimens each, 1 appliance for each phase. Each study’s phase lasted 5 days, had 7 days of interval before the following phase, and corresponded to each evaluated group. Erosive challenges were performed by palatal appliances immersion in 17.6 mL of coca cola soft drink (Coca-Cola®) at room temperature, 3 times per day x 5 minutes x 5 days (75 minutes total). Abrasive challenges were performed after the first and third erosive cycles using an electric toothbrush (Oral-B Vitality D12, P&G Ltad, Louveira, São Paulo, Brazil) during 30 seconds in each specimen associated with the respective dentifrices’ slurry. The volunteers wore the appliances during 12 hours per day, removing it only for meals, oral hygiene, erosive/abrasive tests, and overnight (maintained in containers with humidity).

After the end of each study’s phase, a new profilometer analysis was performed following the previously described protocol. Superimposition of initial and final profiles were performed using MarSurf XCR 20 (Göttingen, Germany) and the dentin loss was quantified.

-Statistical Analysis

Data were analyzed by Repeated Measures Analysis of Variance followed by Fisher’s LSD test (*p*<0.05).

## Results

The wear results for the different tested groups are listed in Figure 1.

All groups presented dentin wear as showed in Table 2. G1 (1.76 ± 0.55) showed the highest values, followed by G2 (1.19 ± 0.42) and G3 (1.29 ± 0.34). G4 (0.93 ± 0.38) and G5 (0.82 ± 0.34) presented the lowest dentin wear among tested groups.

**Table 2:**
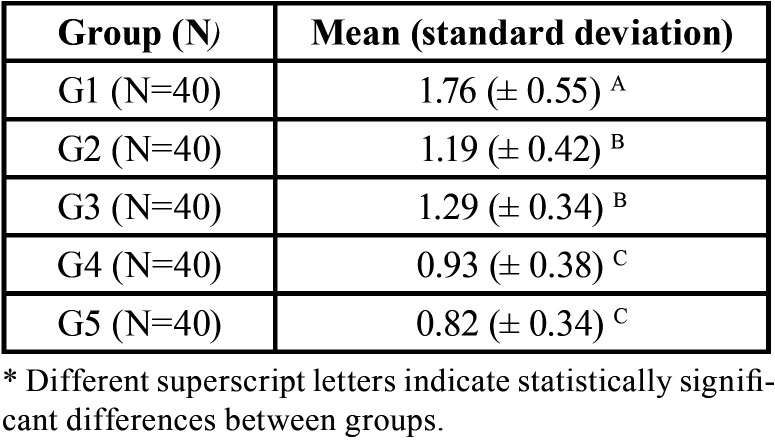
Mean and standard deviation of dentin loss after erosive tooth wear and treated with different dentifrices (Repeated Measures Analysis of Variance followed by Fisher’s LSD test, *p*<0.05).

## Discussion

Many factors may interfere with dental wear. In situ studies are capable of simulate what occur in patient’s mouths, overcoming the limitations with *in vitro* studies (do not fully reproduce intraoral situation) and *in vivo* studies (do not quantify the mineral loss) (16). This said, a crossover protocol is preferred in order to minimize the effect of different volunteers on the final result, resulting in higher statistics reliability (16).

The placebo dentifrice (G1) showed the highest wear rate (1.76 ± 0.55µm), as reported by other authors (17,18), not contributing for an increase in wear resistance when compared with other groups, probably as a result of the absence of a MMPs inhibitor.

Chlorhexidine is a gold-standard inhibitor of MMPs and widely used for *in vitro* and in situ studies (19,20). Chlorhexidine is by chelation of metallic ion blocking the linked of MMPs with these ions and avoid the degradation of collagen, but the CHX also inhibits cathepsins cysteine (19,20). In the present study, CHX was used at 0.012% in a dentifrice (G2), considering that CHX in lower concentration showed effective in inhibit MMPs and minimize the mineral loss in dentin (17).

Fluoride-containing dentifrices (G3) are used as remineralization agent and, although fluorides present an extremely low potential, they inhibit MMPs- 2 and 9 enzymes (21), avoiding the degradation of collagen fibrils and maintaining the DOM. Its action happens through competition with MMPs for Ca2+ and Zn2+ (19,21). In this study represented the second positive control.

Based on the present study’s results, both groups G2 and G3 were able to reduce the dentin wear, respectively 1.19 ± 0.42µm and 1.29 ± 0.34µm, when compared with the placebo dentifrice (1.76 ± 0.55µm). Similar results were reported by another *in vitro* study (17).

It is noteworthy that dentifrices containing chlorhexidine can cause side effects, such as teeth staining and/or loss of taste sensitivity (22).

Proanthocyanidin (PA) consists in a natural, renewable, extract (12), which presents little toxicity (19) and is capable of readily inhibit MMPs and cathepsins cysteine (23), as well as prevent mineral loss through cross-linking action which biomodifies the collagen fibrils of the dentin organic matrix, improving its mechanical proprieties (11,13,24,25).

Based on the present study results, PA containing dentifrices showed the lowest dentin wear when associated (G5) or not (G4) with fluorides, respectively 0.82 ± 0.34µm and 0.93 ± 0.38µm. The association with fluorides might be beneficial because of the increased MMPs inhibition and above-mentioned remineralization capabilities (6,17,26,27). This said, the null hypothesis was rejected.

The protocol chosen was use of devices during the day, due to difficulty collaboration of volunteers for use during the night (16), as also due to the limited remineralization activity during the nighttime (28), and the remineralization intervals consisted of a minimum of 2 hours until the following planned erosion and/or abrasion cycle. The intervals to remineralization adopting during the use were of minimum 2 hours for next erosion and abrasion cycle.

Coca Cola was adopted as the erosive challenge due to its lower pH, potential wear, and wide consumption by the society (29). Bovine teeth were selected as it is similar to human dentin when considering evaluation of MMPs activity (30).

In the present study, dentifrice was chosen as a treatment delivery system since it is easy to implement and widely used by the population. In summary, with this study Proanthocyanidin’s dentifrices and its combination with fluoride dentifrices showed reduce the mineral loss of dentin tissue after erosion and dental abrasion. Based on that, these news formulations of dentifrice can be an interesting choice for patients who present clinical dentin lesions resulting from erosion and abrasion.

## References

[1] Lussi A, Hellwig E, Zero D, Jaeggi T (2006). Erosive tooth wear: diagnosis, risk factors and prevention. Am J Dent.

[2] El Aidi H, Bronkhorst EM, Huysmans MC, Truin GJ (2011). Multifactorial analysis of factors associated with the incidence and progression of erosive tooth wear. Caries Res.

[3] Rochel ID, Souza JG, Silva TC, Pereira AFF, Rios D, Buzalaf MAR (2011). Effect of experimental xylitol and fluoride-containing dentifrices on enamel erosion with or without abrasion in vitro. J Oral Sci.

[4] Ganss C, Schlueter N, Preiss S, Klimek J (2009). Tooth brushing habits in uninstructed adults--frequency, technique, duration and force. Clin Oral Investig.

[5] Kleter GA, Damen JJ, Everts V, Niehof J, Ten Cate JM (1994). The influence of the organic matrix on demineralization of bovine root dentin in vitro. J Dent Res.

[6] Buzalaf MA, Kato MT, Hannas AR (2012). The role of matrix metalloproteinases in dental erosion. Adv Dent Res.

[7] Tjaderhane L, Buzalaf MA, Carrilho M, Chaussain C (2015). Matrix metalloproteinases and other matrix proteinases in relation to cariology: the era of 'dentin degradomics'. Caries Res.

[8] Zarella BL, Cardoso CA, Pela VT, Kato MT, Tjaderhane L, Buzalaf MA (2015). The role of matrix metalloproteinases and cysteine-cathepsins on the progression of dentine erosion. Arch Oral Biol.

[9] Ganss C, Hardt M, Blazek D, Klimek J, Schlueter N (2009). Effects of toothbrushing force on the mineral content and demineralized organic matrix of eroded dentine. Eur J Oral Sci.

[10] Epasinghe DJ, Burrow MF, Yiu CKY (2017). Effect of proanthocyanidin on ultrastructure and mineralization of dentine collagen. Arch Oral Biol.

[11] Castellan CS, Pereira PN, Grande RH, Bedran-Russo AK (2010). Mechanical characterization of proanthocyanidin-dentin matrix interaction. Dent Mater.

[12] Bedran-Russo AK, Pauli GF, Chen SN, McAlpine J, Castellan CS, Phansalkar RS (2014). Dentin biomodification: strategies, renewable resources and clinical applications. Dent Mater.

[13] Liu Y, Chen M, Yao X, Xu C, Zhang Y, Wang Y (2013). Enhancement in dentin collagen's biological stability after proanthocyanidins treatment in clinically relevant time periods. Dent Mater.

[14] Boteon AP, Prakki A, Rabelo Buzalaf MA, Rios D, Honorio HM (2017). Effect of different concentrations and application times of proanthocyanidin gels on dentin erosion. Am J Dent.

[15] Magalhaes AC, Wiegand A, Buzalaf MA (2014). Use of dentifrices to prevent erosive tooth wear: harmful or helpful?. Braz Oral Res.

[16] West NX, Davies M, Amaechi BT (2011). In vitro and in situ erosion models for evaluating tooth substance loss. Caries Res.

[17] Hannas AR, Kato MT, Cardoso Cde A, Magalhães AC, Pereira JC, Tjäderhane L (2016). Preventive effect of toothpastes with MMP inhibitors on human dentine erosion and abrasion in vitro. J Appl Oral Sci.

[18] Nehme M, Jeffery P, Mason S, Lippert F, Zero DT, Hara AT (2016). Erosion Remineralization Efficacy of Gel-to-Foam Fluoride Toothpastes in situ: A Randomized Clinical Trial. Caries Res.

[19] Breschi L, Maravic T, Cunha SR, Comba A, Cadenaro M, Tjäderhane L (2018). Dentin bonding systems: From dentin collagen structure to bond preservation and clinical applications. Dent Mater.

[20] Scaffa PM, Vidal CM, Barros N, Comba A, Cadenaro M, Tjäderhane L (2012). Chlorhexidine inhibits the activity of dental cysteine cathepsins. J Dent Res.

[21] Kato MT, Bolanho A, Zarella BL, Salo T, Tjaderhane L, Buzalaf MA (2014). Sodium fluoride inhibits MMP-2 and MMP-9. J Dent Res.

[22] Frank ME, Gent JF, Hettinger TP (2001). Effects of chlorhexidine on human taste perception. Physiol Behav.

[23] Epasinghe DJ, Yiu CK, Burrow MF, Hiraishi N, Tay FR (2013). The inhibitory effect of proanthocyanidin on soluble and collagen-bound proteases. J Dent.

[24] Bedran-Russo AK, Castellan CS, Shinohara MS, Hassan L, Antunes A (2011). Characterization of biomodified dentin matrices for potential preventive and reparative therapies. Acta Biomater.

[25] Balalaie A, Rezvani MB, Mohammadi Basir M (2018). Dual function of proanthocyanidins as both MMP inhibitor and crosslinker in dentin biomodification: A literature review. Dent Mater J.

[26] Pavan S, Xie Q, Hara AT, Bedran-Russo AK (2011). Biomimetic approach for root caries prevention using a proanthocyanidin-rich agent. Caries Res.

[27] Tang CF, Fang M, Liu RR, Dou Q, Chai ZG, Xiao YH (2013). The role of grape seed extract in the remineralization of demineralized dentine: micromorphological and physical analyses. Arch Oral Biol.

[28] Alencar CR, Mendonca FL, Guerrini LB, Jordão MC, Oliveira GC, Honório HM (2016). Effect of different salivary exposure times on the rehardening of acid-softened enamel. Braz Oral Res.

[29] Reddy A, Norris DF, Momeni SS, Waldo B, Ruby JD (2016). The pH of beverages in the United States. J Am Dent Assoc.

[30] Kato MT, Hannas AR, Leite AL, Bolanho A, Zarella BL, Santos J (2011). Activity of matrix metalloproteinases in bovine versus human dentine. Caries Res.

